# Preventive effects of different protective agents on dentin erosion: An *in vitro* investigation

**DOI:** 10.4317/jced.53129

**Published:** 2017-01-01

**Authors:** Claudio Poggio, Chiara Gulino, Maria Mirando, Marco Colombo, Giampiero Pietrocola

**Affiliations:** 1 Department of Clinical-Surgical, Diagnostic and Pediatric Sciences, Section of Dentistry, University of Pavia, Pavia, Italy; 2 Department of Molecular Medicine, Unit of Biochemistry, University of Pavia, Pavia, Italy

## Abstract

**Background:**

The purpose of this *in vitro* study was to evaluate the preventive effects of different protective agents on dentine erosion, measuring mean percentage weight loss. Dissolution of dentine under erosive challenges caused by soft drinks was analyzed: specimens were weighed following each immersion period, with mean percent weight losses calculated.

**Material and Methods:**

Extracted teeth were sectioned into uniform slabs. Seventy permanent enamel specimens were randomly distributed to seven groups. Initial weights of all dentin specimens were performed. The fluoride pastes Remin Pro, MI Paste Plus, Tooth Mousse, Biorepair, Biorepair Plus and Regenerate were used in this study. A control group was treated just with tap water. The specimens then were immersed in Coca-Cola for a total of 32 min at room temperature. Finally each specimen was dry and weighed. The mass loss was calculated as a percentage of that observed prior the fluoride pastes application. Weight loss data were subjected to Analysis of Variance (One-way ANOVA) followed by Bonferroni’s post hoc tests.

**Results:**

Percent weight loss of specimens exposed to early stages in Coca-Cola showed linear progression with time. Specimen’s application of fluoridated varnishes such as Biorepair or Regenerate, prior immersion in Coca-Cola, significantly protect dentin from demineralization. Otherwise, application of Tooth Mousse or Biorepair Plus increased dentin demineralization starting from 24 min of immersion in Coca-Cola.

**Conclusions:**

Despite the limitations of this study, the protective pastes that showed the less weight loss due to the acidic challenge are Biorepair and Regenerate.

** Key words:**Dentine, erosion, protective agents, soft drinks, toothpastes.

## Introduction

Dental erosion is defined as the loss of tooth substance due to chemical processes not involving bacteria, caused by a variety of intrinsic and extrinsic factors. Intrinsic factors are the result of endogenous acid, generally gastric acids that contact teeth especially in patients suffering from anorexia, bulimia and gastrointestinal disturbances. Extrinsic factors are related to frequent consumption of acidic foodstuffs or beverages and exposure to acidic contaminants in the working environment. The consumption of citric fruit and juices, and industrialized beverages, especially soft drinks, has significantly increased during recent years, and has been associated with an increase in the prevalence of dental erosion. As lifestyles have changed, there has been a 50% increase in consumption of soft drinks over the past few decades, especially among children and adolescents. Dietary changes and inadequate oral hygiene have led to erosion becoming more frequent among young people. The development of erosion involves a chemical process in which the inorganic phase of the tooth is demineralized, thereby reducing the hardness of the tooth substrates. Subsequent abrasive challenges through brushing increase the loss of the tooth substrates (1). The loss of substance by erosion is a dynamic process with periods of demineralization and remineralization. Thus, preventive measures against erosion are required. Dental enamel consists of 95% calcium hydroxyapatite, 4% water, 1% organic mineral. Enamel is organized in prisms: a-prismatic enamel with a thickness of up to 100 microns has been reported to be present at the enamel surface, which is generally more highly mineralized than subsurface. The early stages of dental erosion are characterized by a softening of the enamel surface to a depth of the order of 1-10 microns. Many studies have been carried out to understand the process of enamel demineralization at the early stages, but there are still little known about if these early stages are reversible (2). Biological and chemical factors in the oral environment influence the progress of dental erosion. Saliva provides protective effects by neutralizing and clearing the acids; it is also a source of inorganic ions necessary for the remineralization process (3). Enamel has no spontaneous capability to repair when affected by specific dental pathologies such as caries, abrasions or fractures because it contains no cells (4). Dentine is the tissue underlying the enamel that forms the bulk of the tooth. The dentin matrix is formed by about 45-50 vol% of mineral in the form of a carbonated hydroxyapatite, 30-35 vol% of organic matter, mostly as type I collagen with associated non collagenous protein (5). Apatite in dentin has a much smaller crystallite size, higher carbonates content and is more susceptible to acidic dissolution than enamel apatite. Hence, once the demineralization process involves dentine, its rate will be accelerated (6). Most soft drinks are acidic in nature and exposure to these drinks may result in dental erosion. Professionally applied highly fluoridated varnishes have been proposed as a preventive management of tooth wear which is predominantly caused by acid erosion resulting from the dietary habits of the contemporary lifestyle. The aim of this study *in vitro* was to evaluate the preventive effects of different fluoride varnishes on dentine erosion. Dissolution of dentine resulting from immersion in soft drinks will be measured.

## Material and Methods

Recently extracted maxillary and mandibular primary and permanent human teeth, free of hypo calcification, caries, and macroscopic fractures were carefully cleaned of calculus and other debris. The teeth were previously stored in a 1% Chloramine-T solution (Fisher Chemical, Fair lawn, NJ, USA) consisting of 12% active chlorine diluted in distilled water. For the dentine the crowns were removed at cemento-enamel junction using an Accutom-50 diamond cutter (Accutom Hard Tissue Microtome, Struers, Ballerup, Denmark) under water-cooling. The root surface was treated with a low speed fine-grain diamond bur (Perio-Set, Intensiv, Grancia, Switzerland) under abundant irrigation with Peeso burs n°4 to n°6 (Dentsply Maifeller, Ballaigues; Switzerland) using a contra-angle handpiece. After each instrument, the root canal was irrigated with 5 ml of distilled water. Two transversal sections of 2-mm thickness were obtained from the cervical third of each root using a pre-programmed automatic Accutom-50 diamond cutter (Accutom Hard Tissue Microtome, Struers, Ballerup, Denmark). Each slice was then sectioned in four sections, obtaining a total of four (s1, s2, s3, s4) samples from each root. Dentin specimens were divided among different protective agents and placed into separated containers, with one specimen per container. Samples were then catalogued and stored into distilled water at room temperature. Initial weights of all dentin specimens were performed prior to soft drink immersion. A soft drink (Coca-Cola, Coca-Cola Company, Milano, Italy) was chosen for the demineralization process.

For prevention of dentin erosion, different fluoride pastes (Remin Pro, MI Paste Plus, Tooth Mousse, Biorepair, Biorepair plus, Regenerate) were evaluated. The characteristics, chemical composition and manufacturer of the products tested are reported in  Table 1.

**Table 1:**
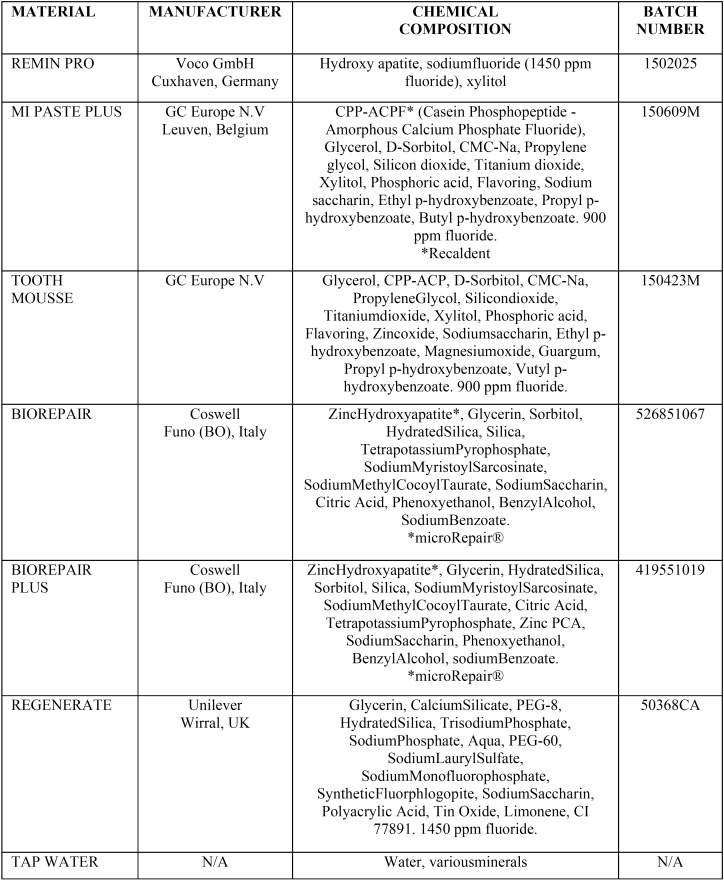
Protective materials used in this study.

The samples were randomly attributed to 7 groups (n= 10). The specimens belonging to group 1 were treated with tap water instead of any fluoride pastes (control). For the specimens of groups 2, 3, 4, 5, 6, protective pastes neat onto the surface of the specimens without brushing for 3 min at 0, 8, 24, 32 h before demineralization with Coca-Cola were applied ( Table 2). Prior to the start of the experiment all specimens were dried on blotting-paper at room temperature for one hour and weighed using a precision balance (Mettler-Toledo, model AE1633, Novate Milanese, Italy, metering accuracy 0.01 mg). No statistically significant differences in term of weight variation before or after fluoride pastes application were found (*p*>0.05, ANOVA with Bonferroni post hoc test). Thereafter, all specimens were simultaneously placed in a pvc pannier which was suspended in a plastic container containing 6 ml of Coca-Cola and immersed for 2 min at room temperature before rinsing with deionized water. Four consecutive intervals of the immersion procedure were carried out for a total of 8 minutes (7). Each specimen was removed from the beverage using tweezers, blotted dry with blotting paper, left undisturbed to dry for 60 minutes, and weighed. The mass loss was calculated as a percentage of that observed prior the fluoride pastes or tap water (control) application (mass set to 0%). Continuous data were expressed as means and standard deviations. Weight loss data were subjected to Analysis of Variance (One-way ANOVA) followed by Bonferroni’s post hoc tests. Analyses were performed using Prism 4.0 (GraphPad). Two-tailed *P* values of 0.05 were considered statistically signiﬁcant.

**Table 2:**
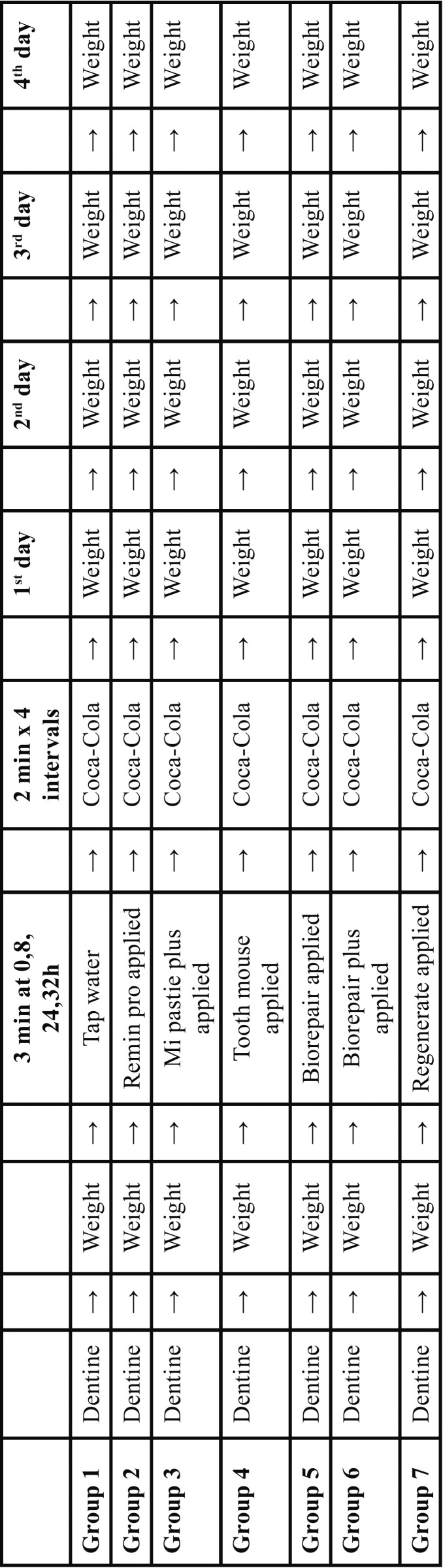
Diagram of the study design.

## Results

**Figure 1:**
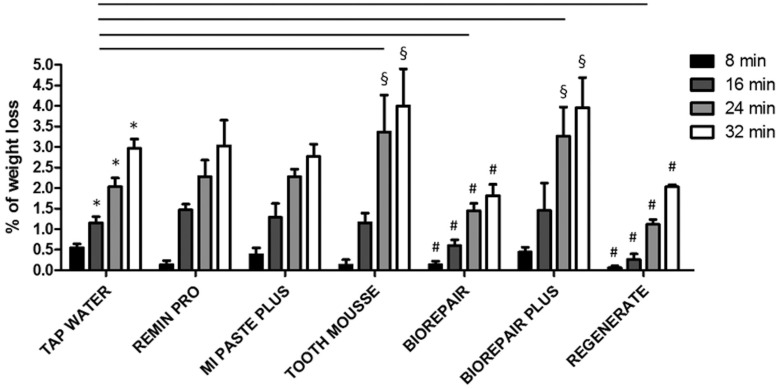
Relation between weight loss of enamel and dentine of teeth specimens and time. The mass loss was calculated as a percentage of that observed prior the fluoride pastes or water (control) application (mass set to 0%). The reported data are the mean values (+/- SD). Symbols (*, #, §) indicate statistically significant differences (*p* < 0.05) as determined by repeated-measures one-way ANOVA with a Bonferroni’s post hoc tests.

Loss of dentine after long period (several days) exposure to different non-alcoholic drinks such as Coca-Cola has been well documented (8). In our experimental conditions we take in considerations loss of dentin by early stages exposure (8 to 32 min) in soft-drink: Coca-Cola was able to cause loss of dentine already starting form 8 min exposure (0.546 ± 0.16) (group 1). Statistically significant (*p*< 0.05) weight loss of dentine increased linearly with time, after 16 min (1.22 ± 0.31), 24 min (2.03 ± 0.42) and 32 min (3 ± 0.42). In order to evaluate if professionally applied highly fluoridated varnishes have preventive management on dentin erosion caused by Coca-Cola, the samples belonging to group 2, 3, 4, 5 and 6 were treated with different fluoride pastes: Remin Pro, MI Paste Plus, Tooth Mousse, Biorepair, Biorepair plus and Regenerate respectively, before immersion in Coca-Cola ( Table 2). As shown in figure 1, Remin Pro and MI Paste Plus, showed a similar trend in terms of demineralization to that observed in control group 1 (*p*> 0.05). The specimens treated with Biorepair or Regenerate, showed significantly lowest mean percent weight loss compared to the control group 1 (*p* < 0.05) for all the considered times (Fig. 1). Otherwise, the specimens treated with Tooth Mousse or Biorepair Plus, showed statistically significant higher erosivity than the control group 1 (*p* < 0.05) since the third immersion in Coca-Cola (24 min to 32 min) (Fig. 1).

## Discussion

Dental erosion is a relatively new risk factor for dental health, introduced by today’s lifestyle (9). Dietary changes and inadequate oral hygiene have led to enamel erosion becoming more frequent among young people. Demineralization of teeth by erosion is caused by frequent contact between the tooth surface and acids, due to the increased consumption of acid drinks as soft drinks, sport drinks, fruit juices and fruit teas (10). The occurrence of erosion lesions in deciduous dentition shows the need to give parents guidance to provide their children a diet without too many acidic products (11).

The hardness of enamel significantly decreased after 8 min immersion in a cola drink (7). The thickness of the softened layer is depend on the chemical composition of the erosive drink; drinks which rapidly dissolve surface enamel also diffuse further into the enamel bulk in a given timeframe and can cause the greatest subsurface softening (12). The characteristics of erosion may be due to several factors, including the chemical properties of the erosive medium and the frequency and method of contact between acid and tooth. Biological and chemical factors in the oral environment influence the progress of dental erosion. Saliva provides protective effects by neutralizing and clearing the acids; it is also a source of inorganic ions necessary for the remineralization process (13); this is the reason why patients with diminished salivary flow are more exposed to dental erosion and decay (13,14).

The decrease in surface hardness that accompanies the early stages of enamel erosion by dietary acids is well recognized, and its quantification by microindentation has been employed as a mean of assessing the relative efficacy of anti-erosion treatments such as toothpastes and mouthrinses. The utility of NaF, whether delivered as a simple solution or from toothpaste, to reduce surface roughening and bulk tissue loss has been clearly demonstrated using white light interferometry (15). Enamel and dentin have no cells and thus no ability to spontaneously regenerate. Any deterioration is therefore biologically irreversible. Therefore, it seems reasonable to search for effective agents for prevention or repair of these erosions. Despite the wide use of fluoride, new agents to control erosion have been proposed and recommended for individual or professional-based application.

For prevention of dentin erosion, different protective agents (Remin Pro, MI Paste Plus, Tooth Mousse, Biorepair, Biorepair plus, Regenerate) were evaluated. The characteristics, chemical composition and manufacturer of the products tested are reported in  Table 1. Remin Pro is toothpaste containing Hydroxyapatite, sodium fluoride (1450 ppm fluoride), and xylitol. Tooth Mousse and MI Paste Plus are remineralizing agents based on casein phosphopeptide-stabilized amorphous calcium phosphate complexes, CPP-ACP and casein phosphopeptide-stabilized amorphous calcium fluoride phosphate complexes, CPP-ACFP. Casein is the major protein group found in milk and accounts for approximately 80% of the total protein (16). The ability of casein to stabilize calcium and phosphate ions resides in sequences that can be released as small peptides (case in phosphopeptides) by partial enzymic digestion. These complexes, CPP-ACP and CPP-ACFP, have been incorporated into dental cream and stabilize and deliver bioavailable calcium, phosphate and fluoride ions (17). The softened enamel caused by soft drink, which represented the early stage of erosion, became hardened after four application of a CPP-ACP paste (7). Biorepair® is the first microRepair® based toothpaste that can penetrate enamel and dentin micro-scratches, binding to and chemically repairing microabrasions. Thanks to the presence of microRepair®, Biorepair® protects and repairs as well. MicroRepair® consists of particles constituted by Hydroxyapatite whose composition is very similar to that of tooth enamel. This similarity gives microRepair® the biomimetic properties to integrate microparticles with enamel and dentin, with consequent mineralising and restorative action. Biorepair Plus (Coswell S.p.A., Bologna, Italy) is a fluoride free toothpaste made of hydroxyapatite nanocrystals, which have been introduced because of their excellent biological properties, lack of toxicity and inflammatory and immunological responses. The hydroxyapatite microparticles are completely identical to the mineral that forms dentine and enamel. In the case of the enamel, the microparticles action takes place via their ability to bond to natural tissues, thus filling microgaps in the enamel (13,18). Regenerate is a new calcium silicate and sodium phosphate salts (monosodium phosphate and trisodium phosphate) toothpaste containing 1450ppm fluoride (added as sodium mono-fluorophosphate). It has been developed to provide enhanced enamel health benefits (19).

The common thread with all the products in this study was a paste containing fluoride. A significant challenge was the differing composition and concentrations of fluoride in the paste. But there was no real opportunity to overcome this, as we were reliant upon commercial products. Fluoride concentration varied considerably between the protective agents. The results demonstrated that the highly concentrated fluoride agents protect the enamel in this laboratory model. The highest concentrations of fluoride are present in Remin Pro (1450 ppm) and in Regenerate (1450ppm) whilst MI Paste plus and Tooth Mousse have considerably less (900ppm). It is extremely challenging to assess the influence of the protective agents on erosion or erosion-abrasion.

Percent weight loss of specimens exposed to early stages in Coca-Cola showed linear progression with time. Group 1 is the control group, just treated with tap water in order to investigate the demineralization process in Coca-Cola. Pastes which recorded the lowest values of weight loss after immersion in Coca Cola are Biorepair and Regenerate: this means that these pastes are resistant to acid attack effectively. The influence of these toothpastes on the prevention of demineralization observed in the present study would be clinically beneficial. As regards the accuracy of the results, the group 5 (Biorepair) and the group 7 (Regenerate) respond in a uniform manner to measurement: as can be seen from figure 1 the results obtained are concentrated in a narrow range of values. Compared to the control group 1, the results of the specimens treated with Biorepair and with Regenerate are statistically significant (*p* < 0.05). Groups 2 and 3 ( Remin Pro and MI Paste Plus) obtained results that are similar to those of group control: the specimens treated with these pastes didn’t show significant difference than group 1 (*p* > 0.05). Finally the specimens belonging to group 4 and 6 (Tooth Mousse and Biorepair Plus) showed statistically significant higher erosivity than the control group (*p* < 0.05) since the third immersion in soft-drink. Figure 1 clearly shows the high weight loss values.

## Conclusions

One of the potential limitations of the study was that our *in vitro* conditions did not reproduce *in vivo* conditions, which we did not consider in our analysis. In the oral environment, host factors (such as the mineral concentration of the tooth, and the pellicle and plaque formation) can influence the progression of demineralization (20-22). Salivary factors, such as the salivary flow rate, composition and buffering capacity, might exert protective action on dental surface (4,20,23).

In conclusion, despite the limitations of this study, the protective pastes that showed the less weight loss due to the acidic challenge are Biorepair and Regenerate. Remin Pro and MI Paste Plus although reached higher percent weight loss hardness values than the previous materials, otherwise application of Tooth Mousse or Biorepair Plus didn’t protect dentin from demineralization cau-sed by soft drink from the third immersion in Coca-Cola. This difference in step height might reflect the effect of fluoride concentration and formulation while their rapid release of fluoride in few hours could have an additional effect. Although not great, it does appear that ﬂuoride can afford protection to enamel and dentine against erosion (wear dentine).

To conclude, with the caution that must be afforded to extrapolating all data generated *in vitro* to clinical meaning, this study indicates as to how susceptible dentine is to erosion by soft drinks. Toothpastes appeared to afford protection against erosion rather than accelerating dentin loss but the composition of the pastes affect the results in different way.
